# The effects of depression and use of antidepressive medicines during pregnancy on the methylation status of the *IGF2 *imprinted control regions in the offspring

**DOI:** 10.1186/1868-7083-3-2

**Published:** 2011-10-26

**Authors:** A Soubry, SK Murphy, Z Huang, A Murtha, JM Schildkraut, RL Jirtle, F Wang, J Kurtzberg, W Demark-Wahnefried, MR Forman, C Hoyo

**Affiliations:** 1 Duke Cancer Institute, Duke University Medical Center, Durham, NC, USA; 2 Dept. of Obstetrics and Gynecology, Duke University Medical Center, Durham, NC, USA; 3 Dept. of Community and Family Medicine, Duke University Medical Center, Durham, NC, USA; 4 Dept. of Radiation Oncology, Duke University Medical Center, Durham, NC, USA; 5 Dept. of Pediatrics, Duke University Medical Center, Durham, NC, USA; 6 Dept. of Nutrition Sciences, University of Alabama at Birmingham, Birmingham, AL, USA; 7 Dept. of Epidemiology, M.D. Anderson Cancer Center, Houston, TX, USA

**Keywords:** antidepressants, depression, pregnancy, *IGF2*, offspring, race

## Abstract

*In utero *exposures to environmental factors may result in persistent epigenetic modifications affecting normal development and susceptibility to chronic diseases in later life. We explored the relationship between exposure of the growing fetus to maternal depression or antidepressants and DNA methylation at two differentially methylated regions (DMRs) of the imprinted *Insulin-like Growth Factor 2 *(*IGF2*) gene. Aberrant DNA methylation at the *IGF2 *and neighboring *H19 *DMRs has been associated with deregulated *IGF2 *expression, childhood cancers and several chronic diseases during adulthood. Our study population is comprised of pregnant mothers and their newborns (n = 436), as part of the Newborn Epigenetics Study (NEST). A standardized questionnaire was completed and medical record data were abstracted to ascertain maternal depression and antidepressive drug use. DMR methylation levels in umbilical cord blood leukocytes were quantified using pyrosequencing. From the 436 newborns, laboratory data were obtained for 356 individuals at the *IGF2 *DMRs, and for 411 individuals at the *H19 *DMRs; about half of each group was African American or Caucasian. While overall no association between depression and methylation profiles was found, we observed a significant hypermethylation of the *H19 *DMRs in newborns of African American (n = 177) but not Caucasian (n = 168) mothers who reported the use of antidepressive drugs during pregnancy (β = +6.89, p = 0.01). Of note, our data reveal a race-independent association between smoking during pregnancy and methylation at the *IGF2 *DMR (+3.05%, p = 0.01). In conclusion, our findings suggest a race-dependent response related to maternal use of antidepressants at one of the *IGF2 *DMRs in the offspring.

## Introduction

Epigenetic mechanisms are important for regulating gene expression and differentiation during early life. Recent studies have highlighted the possible impact of environmental factors on epigenetic characteristics during development. *In utero *exposure to chemicals, nutrition, or social factors may change the methylation status at CpG-rich regions of gene promoter regions, causing permanent modification of gene expression patterns [1-3]. Such alterations may lead to increased risk of chronic diseases, including mental disorders, diabetes, cardiovascular diseases and cancer [4-6].

Maternal depression, and associated drug use are common exposures to the developing fetus. The prevalence of depression in pregnant women is greater than ten percent [7], and the rate of prescriptions for mood regulators reported among pregnant women in the U.S. has increased threefold, from 1998 to 2005 [8]. Co-occuring adverse factors include: inadequate nutrition intake or insufficient weight gain, and cigarette smoking [9]. It has been shown that *in utero *exposure to maternal depression adversely affect fetal growth [10,11], fetal neurobehavioral development, or childhood behavior [12-15]. Exposure to SSRIs (Selective Serotonin Reuptake Inhibitors) has been associated with congenital malformations [16-20], respiratory distress, or neurobehavioral symptoms in newborns [21,22]. As a consequence, treatment of psychiatric disorders during pregnancy is controversial; the fetus is either exposed to the psychotropic drugs or to the disease itself.

The biological mechanisms behind the adverse fetal developmental consequences of antidepressant use of the mother or exposure to maternal depression are unclear. Evidence suggests that mood disorders or antidepressant medicines are associated with modulation of epigenetic regulation [23]. Adverse social environments can induce altered DNA methylation at the promoter of the glucocorticoid receptor gene in the rat hippocampus [24,25,2]. Aberrant methylation was detected at the promoter regions of the *rRNA *gene in patients who were suicidal [26]; and suicide victims with a history of childhood abuse showed a decreased level of glucocorticoid receptor mRNA and an increased site-specific methylation at the promoter of the neuron-specific glucocorticoid receptor (*NR3C1*) gene [27]. Similar aberrant methylation patterns were found in cord blood of newborns from mothers suffering depression or anxiety during their third trimester of pregnancy [28].

These observations prompted us to search for promoters of other genes where methylation patterns might also be affected by adverse socio-environmental factors during pregnancy. The *Insulin-like growth factor 2 *(*IGF2*) gene is an epigenetically regulated imprinted gene with important roles during embryonic and fetal growth. *IGF2 *imprinting and expression are regulated at least in part by methylation of two differentially methylated regions (DMRs), the DMR upstream of the *IGF2 *promoters (*IGF2 *DMR) and the DMR upstream of the adjacent *H19 *gene (*H19 *DMR). In most normal tissues, the *IGF2 *and *H19 *DMRs are expected to be methylated at only one of the parentally inherited alleles [29-31]. It has been demonstrated that prenatal exposures to adverse social or nutritional environments result in aberrant *IGF2 *methylation that could lead to deregulated expression, including loss of imprinting (LOI) or activation of expression from the normally silent maternal allele [1]. A direct causal relationship has recently been shown in ewes: periconceptional undernutrition resulted in a decrease in methylation at the CTCF-binding site upstream of *H19 *in the offspring [3], corresponding to the *H19 *DMR in human. We and others have hypothesized that the methylation profile at the imprint regulatory elements (or DMRs) could serve as a biosensor and adverse environmental conditions acquired early in life could be archived in the epigenome [32,33].

In this study, we evaluate whether *in utero *exposure to maternal depression or intake of antidepressants is associated with variation in methylation at the two regulatory DMRs of the *IGF2 *imprinted domain.

## Materials and methods

### Study subjects and data collection

Study participants are part of the Newborn Epigenetics Study (NEST) at Duke University, a multi-ethnic birth cohort. Pregnant women were recruited between April 2005 and June 2008 from prenatal clinics serving Duke Obstetrics and Durham Regional Hospital Obstetrics facilities, in North Carolina. Recruitment strategies have been detailed in a previous study; in the first 200 subjects smoking mothers were targeted [34]. Eligibility criteria were: age ≥18 years, English speaking, being pregnant (no gestation age criteria were applied), and intending to use one of the two obstetric facilities for the index pregnancy to ensure access to birth outcomes data and umbilical cord blood. During the three-year period, 838 pregnant women who met the eligibility criteria were identified through appointment logs and asked to enroll in the study. Of these women, 690 (82.3%) agreed to participate in the study and were followed throughout the remainder of their pregnancy. A standardized questionnaire was administered at enrollment. Questions were either self- or interviewer-completed, and the data were further verified from medical records. Questionnaires included socio-demographic data (such as race and education), morbidity (including depression), lifestyle characteristics (such as cigarette smoking, and alcohol use), and vitamin supplementation. Along with other medical conditions, pregnant women were asked to respond "yes" or "no" to the question: "What describes any ailment you may have?"; depression was one of the 15 conditions. Clinical charts were used to verify depression. The use and type of antidepressants, mother's age, birth weight and gender of the baby were abstracted from medical records using the standardized chart abstraction form. Antidepressants fell into the following categories: Selective Serotonin Reuptake Inhibitors (SSRIs), such as sertraline (Zoloft), fluoxetine (Prozac), escitalopram (Lexapro), paroxetine (Paxil), citalopram (Celexa); Serotonin-norepinephrine reuptake inhibitors (SNRIs), such as venlafaxine (Effexor); Tricyclic antidepressants (TCAs), such as Amitriptyline HCl (Elavil); Serotonine Antagonist and Reuptake Inhibitors (SARIs) (Trazadone); and bupropion (Wellbutrin). The type of antidepressant use was diverse, but most were SSRIs (72%). Offspring anthropometric measurements were abstracted at delivery. The analysis focused on the first 436 participants, among whom methylation analyses had been completed for at least one of the two DMRs. The study protocol was approved by the Institutional Review Boards of the University of Texas - M.D. Anderson Cancer Center, and Duke University.

### Specimen collection

At delivery, the umbilical vein was punctured and cord blood samples were collected within minutes of delivery in a vacuum blood collection tube, coated with K_3_EDTA. The tubes were inverted gently to mix the anticoagulant with the blood and transported within 12 hours to the laboratory. After centrifugation the leukocyte-containing buffy coat was isolated and stored at -80°C. Genomic DNA was extracted using Gentra Puregene Reagents (Qiagen, Valencia, CA).

### DNA methylation analysis

The *IGF2 *and *H19 *DMRs were analyzed by pyrosequencing. The *IGF2 *DMR includes three CpG dinucleotides upstream of exon 3 (chr 11p15.5; CpG site 1: 2 109 519; CpG site 2: 2 109 516; and CpG site 3: 2 109 500; NCBI Human Genome Build 37.1) [35]. The region studied for the *H19 *DMR encompasses four dinucleotides located upstream of the *H19 *gene (chr 11p15.5; CpG site 1: 1 964 261, CpG site 2: 1 964 259, CpG site 3: 1 964 257, and CpG site 4: 1 964 254; NCBI Human Genome Build 37.1), which is within one of the six CCCTC (CTCF) binding sites [36]. Genomic DNA was treated with sodium bisulfite, which converts unmethylated cytosines to uracils while leaving methylated cytosines unchanged. The samples were amplified by PCR and methylation was quantified in duplicate. Pyrosequencing was performed using a Biotage Pyromark MD pyrosequencing instrument (Qiagen, Valencia, CA). Usually *IGF2 *only expresses its paternally inherited allele, corresponding to a theoretical methylation percentage of 50. When both alleles are methylated the methylation status is expected to be higher than 50%. Although the fact that *IGF2 *is a well studied imprinted gene whose epigenetic profile should be similar across all cell types, we verified the *IGF2 *and *H19 *DMR methylation profiles in DNA from different cell fractions from umbilical cord blood and found no differences across the cell types [37]. We finally analyzed cord blood DNA of the first 436 participants, and obtained experimental data for 356 participants at the *IGF2 *DMR, and for 411 participants at the *H19 *DMR. The CV for the laboratory assays was 0.14 for the *IGF2 *DMR and 0.13 for the *H19 *DMR. The measurements at the three and four CpGs of *IGF2 *DMR and *H19 *DMR, respectively, are highly correlated. The pair-wise Pearson correlation coefficients for the three CpGs at the *IGF2 *DMR are all 0.80, and the pair-wise Pearson correlation coefficients for the four CpGs at the *H19 *DMR are between 0.92 and 0.96. We also ran control assays to validate our results. We used defined mixtures of fully methylated and fully unmethylated bisulfite modified DNAs, and ran the assays in triplicate. Standard deviations varied between 0.27% and 1.89% at the *IGF2 *DMR, and between 0.47% and 2.55% at the *H19 *DMR. R-squared values were 0.99 for both tests, at the *IGF2 *and the *H19 *DMRs.

### Statistical methods

Variables were defined as follows: depression (yes/no), antidepressive drugs (yes/no), smoking status (yes/no), marital status (living with partner or married *versus *single), age (< 30 *versus *≥30), race (African American *versus *non-African American), pre-pregnancy maternal BMI (≤30 kg/m^2 ^*versus *> 30 kg/m^2^), birth weight of the baby (< 2.5 kg *versus *≥2.5 kg), and at least a college graduate (yes/no). Chi Square tests were used to compare depression and intake of antidepressant medicines within different subgroups of pregnant women (Table 1). Methylation levels were distributed normally in the groups studied (verified by using the Kolmogorov-Smirnov test). In Table 2, Student's t-tests were computed to test for significant differences in the methylation means. We further assessed the effect of exposure to antidepressants and depression during development on the methylation levels of *IGF2*; *IGF2 *and *H19 *DMRs were analyzed separately. We used multiple regression models, separately for each exposure, while controlling for potential confounding variables (see Table 3). The potential confounders included the characteristics found to be associated with depression/antidepressants and methylation, with p-values < 0.20 (see Tables 1 and 2) [38]. DNA methylation was the dependent variable and the independent variables were included as described above.

**Table 1:** Distribution of depression and intake of antidepressants in 436 study participants by socio-demographic characteristics in the Newborn Epigenetics Study (NEST), NC, 2005-2008

Socio-demographic data				Depression			Antidepressant use		
	Subgroup	n^1^	%^1^	n	%	*p-value*	n	%	*p-value*
**Total:**		436	100	66	15.1		44	10.1	
**Maternal age:**	< 30	240	55.1	44	18.3	0.04	20	8.3	0.18
	≥30	196	44.9	22	11.2		24	12.2	
**Marital status:**	living with partner	276	63.3	38	13.8	0.30	30	10.9	0.48
	single	160	36.7	28	17.5		14	8.8	
**Race**^2^**:**	African American	215	49.3	33	15.4	0.95	14	6.5	0.01
	Caucasian	193	44.3	30	15.5		28	14.5	
**College graduate:**	no	284	65.1	55	19.4	0.0004	31	10.9	0.45
	yes	151	34.6	10	6.6		13	8.6	
**Antidepressant use:**	no	392	89.9	46	11.7	< 0.0001			
	yes	44	10.1	20	45.5				
**Depression:**	no	370	84.9				24	6.5	< 0.0001
	yes	66	15.1				20	30.3	
**BMI:**	< 18	13	3.0	4	30.8	0.14	2	15.4	0.54
	18- < 25	189	43.4	24	12.7		13	6.9	
	25-30	82	18.8	13	15.8		10	12.2	
	> 30	111	25.5	14	12.6		14	12.6	
**Smoking 1 year before**	non-smoker	305	70.0	46	15.1	0.92	30	9.8	0.82
**pregnancy:**	smoker	123	28.2	19	15.4		13	10.6	
**Smoking during**	non-smoker	347	79.6	37	10.7	< 0.0001	29	8.4	0.02
**pregnancy:**	smoker	88	20.2	29	33.0		15	17.1	
**Birth weight:**	< 2500 g	59	13.5	10	16.9	0.80	10	15.2	0.11
	≥2500 g	369	84.6	54	14.6		34	9.2	
**Gender of newborn:**	male	221	50.7	35	15.8	0.60	19	8.6	0.24
	female	207	47.5	29	14.0		25	12.1	

^1 ^The sum of the numbers (or percentages) for each characteristic are not always 436 (or 100), because some data were missing.

^2 ^Other races were: Asian (n = 7), Native American (n = 2), or not listed (n = 13).

**Table 2:** DNA methylation at *IGF2 *DMR and *H19 *DMR sites by socio-demographic characteristics

		*IGF2 *DMR mean methylation% n = 356 (SD)	Δ (p-value)	*H19 *DMR mean methylation% n = 411 (SD)	Δ (p-value)
**Total:**		47.45 (6.89)		60.09 (7.89)	
**Maternal age:**	< 30	47.43 (7.29)	**+0.05 **(0.95)	60.01 (7.44)	**+0.18 **(0.81)
	≥30	47.48 (6.40)		60.19 (8.42)	
**Marital status:**	living with partner	47.10 (6.59)	**+0.97 **(0.20)	59.48 (7.59)	**+1.63 **(0.05)
	single	48.07 (7.37)		61.11 (8.30)	
**Race^1^:**	African American	47.62 (7.96)	**-0.52 **(0.49)	61.00 (7.90)	**-1.80 **(0.03)
	Caucasian	47.10 (5.60)		59.20 (7.60)	
**College graduate:**	no	47.72 (7.04)	**-0.73 **(0.34)	60.73 (8.07)	**-1.83 **(0.03)
	yes	46.99 (6.61)		58.90 (7.45)	
**Antidepressant use:**	no	47.44 (7.06)	**+0.17 **(0.85)	60.12 (7.63)	**-0.25 **(0.87)
	yes	47.61 (5.07)		59.87 (9.98)	
**Depression:**	no	47.21 (6.65)	**+1.52 **(0.13)	60.18 (8.00)	**-0.07 **(0.95)
	yes	48.74 (8.00)		60.11 (7.64)	
**BMI:**	≤ 30	47.81 (7.10)	**-0.71 **(0.41)	59.51 (7.33)	**+1.81 **(0.07)
	> 30	47.10 (7.01)		61.32 (9.02)	
**Smoking 1 year **	non-smoker	47.69 (7.15)	**-0.94 **(0.24)	60.24 (7.90)	**-0.53 **(0.54)
**before pregnancy:**	smoker	46.75 (6.25)		59.71 (7.00)	
**Smoking during **	non-smoker	46.82 (5.84)	**+3.05 **(0.01)	60.07 (7.83)	**+0.14 **(0.88)
**pregnancy:**	smoker	49.87 (9.62)		60.22 (8.22)	
**Birth weight:**	< 2500 g	47.57 (8.24)	**-0.14 **(0.91)	58.96 (5.32)	**+1.34 **(0.15)
	≥2500 g	47.43 (6.67)		60.30 (7.58)	
**Gender of newborn:**	male	47.05 (5.86)	**+0.80 **(0.28)	59.67 (7.93)	**+0.85 **(0.28)
	female	47.85 (6.63)		60.52 (7.95)	

^1 ^Only the data from children born to Caucasian and African American women are included; numbers of other races were too low.

**Table 3:** Multiple linear regression analyses: DNA methylation at *IGF2 *and *H19 *DMR in the offspring in relation to the use of antidepressants, depression, and race of the mother

	*IGF2 *DMR n max.^1 ^= 356			*H19 *DMR n max. ^1 ^= 411		
	**β**	**SE**	**p**	**β**	**SE**	**p**

**Model 1^2^:** Antidepressant use	**-0.47**	1.28	0.71	**+0.54**	1.40	0.70
**Model 2^2^:** Depression	**+0.72**	1.06	0.50	**+0.25**	1.25	0.84
**Model 3^2^, interaction with race:** Antidepressant use Race (being African American) Antidepressants × Race (African American)	**-1.63** **+0.40** **+3.58**	1.55 0.92 2.71	0.29 0.66 0.19	**-2.38** **-0.01** **+9.18**	1.67 1.17 2.95	0.16 0.99 0.002

**^1 ^**Number included in the statistical analyses, this may vary slightly by missing variables in the different models.

**^2 ^**At *IGF2 *DMR: adjusted for age, race, education, and smoking during pregnancy.

**^2 ^**At *H19 *DMR: adjusted for age, race, smoking during pregnancy, marital status, education, BMI, and birth weight.

The useful laboratory data obtained for *IGF2 *DMR (n = 356) and *H19 *DMR (n = 411) were included in our linear regression analyses. From our original cohort we retained 35 of the 44 mothers taking antidepressants (23 Caucasians, 10 African Americans, and 2 other/missing) and 56 of the 66 depressed mothers (26 Caucasians, 27 African Americans, and 3 other/missing) at the *IGF2 *DMR site; we retained 43 of the 44 mothers taking antidepressants (27 Caucasians, 14 African Americans, and 2 other/missing) and 65 of the 66 depressed mothers (29 Caucasians, 33 African Americans, and 3 other/missing) at the *H19 *DMR methylation data. The final number of observations used by the statistical program varied depending on some missing variables included in the models, e.g. 331 (159 Caucasians + 172 African Americans) observations were used for our analysis at the *IGF2 *DMR site, and 345 (168 Caucasians + 177 African Americans) observations were used at the *H19 *DMR site (see Table 4). The distributions of all variables used in our models, in each subgroups, were similar to the distributions in our whole cohort; meaning that missing values are likely at random. All statistical analyses were conducted in SAS v9.2 (SAS Institute Inc., Cary, NC).

**Table 4:** Linear regression analysis: DNA methylation at *IGF2 *and *H19 *DMRs in the offspring in relation to maternal depression and the use of antidepressants, in African American and in Caucasian participants

	*IGF2 *DMR						*H19 *DMR					
	Caucasian **n**^1 ^**= 159**			**African American n**^1 ^**= 172**			Caucasian **n**^1 ^**= 168**			**African American n**^1 ^**= 177**		
	**β**	**SE**	**p**	**β**	**SE**	**p**	**β**	**SE**	**p**	**Β**	**SE**	**p**

**Model 1^2^:** Antidepressant use	**-1.41**	1.27	0.27	**+1.63**	2.58	0.53	**-2.04**	1.67	0.22	**+6.89**	2.53	0.01
**Model 2^2^:** Depression	**+1.10**	1.26	0.38	**+0.29**	1.69	0.86	**+0.15**	1.70	0.93	**+0.07**	1.89	0.97

^1 ^Number of observations in the statistical analysis.

**^2 ^**At *IGF2 *DMR: adjusted for age, education, and smoking during pregnancy.

**^2 ^**At *H19 *DMR: adjusted for age, smoking during pregnancy, marital status, education, BMI, and birth weight.

## Results

### Socio-demographic characteristics of the pregnant women and corresponding methylation status at the *IGF2 *DMR and the *H19 *DMR in the offspring

The distribution of the socio-demographic characteristics of women and their infants are presented in Table 1. The majority of the study population reported their race as African American (49.3%) or Caucasian (44.3%). Other races included Native Americans (0.5%), Asians (1.6%), or "another race" not listed (2.9%); 1.4% was missing. Gestational age at enrollment ranged from 19 to 42 weeks (mean = 38.1 weeks, SD = 2.5). Over half the study population was under 30 years old (55.1%). Fifteen percent of the women reported being depressed during pregnancy and 10% took antidepressive medicines during the pregnancy. Because smoking mothers were targeted in the first half of participants [34], approximately 20% of the women in the study reported smoking during pregnancy. Over 25% were obese (BMI > 30 kg/m^2^) and another 18% were overweight (25 ≤ BMI ≤ 30 kg/m^2^) before pregnancy. A little over 13% of the newborns had a low birth weight and the numbers of male and female newborns were about equal.

We conducted bivariate analyses to evaluate potential confounders for depression and the use of antidepressants. Depression was most frequently reported by women < 30 years of age (p = 0.04), without a college degree (p = 0.0004), and reporting smoking during pregnancy (p = 0.0001). Smoking was also associated with the intake of antidepressants (p = 0.02) and depression (p < 0.0001). The use of antidepressants was more than twice as high in Caucasian mothers compared to African American mothers (14.5% *versus *6.5%, p = 0.01) (Table 1).

Table 2 shows the average methylation percentage: 47.45% at the *IGF2 *DMR and 60.09% at the *H19 *DMR. At both regions, we found no significant differences in mean methylation percentages based on maternal antidepressant use or depression during pregnancy. Smoking during pregnancy was associated with a higher mean methylation percentage at the *IGF2 *DMR (+3.05%, p = 0.01). The mean methylation percentages at the *H19 *DMR sites were higher in infants born to single mothers, African American mothers, mothers without a college degree, and mothers with a high pre-pregnancy BMI. The respective elevation in methylation levels were in the same range: +1.63% (p = 0.05), +1.80% (p = 0.03), +1.83% (p = 0.03), and +1.83% (borderline significant with p = 0.07) (see Table 2).

### Assessment of the impact of maternal depression and use of antidepressants on the methylation profile at the *IGF2 *DMR and *H19 *DMR in the offspring

Multiple regression analyses of DNA methylation at the *IGF2 *DMR site (n = 356) and *H19 *DMR (n = 411) in relation to the use of antidepressants and depression are shown in Table 3. We did not detect a relationship between the methylation status of the offspring and maternal depression, or the intake of antidepressive medicines, at either of the two regulatory elements (models 1-2, Table 3). Further analysis revealed a significant interaction between the use of antidepressants and being African American: β-coefficient was +9.18 at the *H19 *DMR (p = 0.002) (model 3, Table 3). Also a positive, but insignificant, β-coefficient was noted at the *IGF2 *DMR (β = +3.58, p = 0.19) for African Americans exposed to antidepressants. Other possible interactions were also verified, but no statistically significant interactions were seen between antidepressive medicines or depression and education, BMI, marital status, smoking, or age of the mother (not shown).

**Figure 1:**
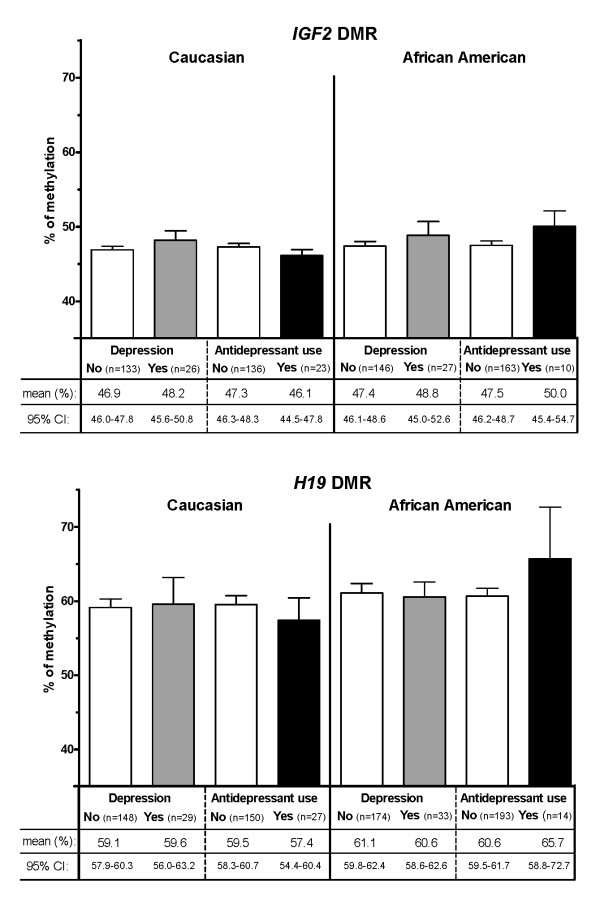
**DNA methylation profiles at *IGF2 *DMR and *H19 *DMR in newborns by race and depression of the mother or the use of antidepressants during pregnancy**.

In order to further explore the interaction between race and antidepressants, we stratified our analysis by race and included only Caucasians and African Americans. We found that exposure to antidepressants among African American newborns was associated with a high methylation profile at *H19 *DMR (β = +6.89, SE = 2.53, p = 0.01) (model 1, Table 4), corresponding to an increase of 5% in methylation mean if unadjusted; from 60.6% (95% CI: 59.6 - 61.7) to 65.7% (95% CI: 58.8 - 72.7) (Figure 1). In contrast, in Caucasian newborns, we detected a rather opposite, but non-significant effect (β = -2.04, SE = 1.67, p = 0.22).

## Discussion

In this study we examined a previously uncharacterized effect of maternal depression and antidepressive medication use during pregnancy on two *IGF2 *differentially methylated regions in the offspring. Maternal depression was not found to independently affect the offspring's methylation status at any of the imprint regulatory regions evaluated. Our data suggest a race-specific influence of the use of antidepressive drugs on the methylation outcomes at the DMR upstream of *H19*, but not significantly at the *IGF2 *DMR.

Over the years, a rapid increase in antidepressant use in pregnancy has been reported [39,8], whereas SSRIs are known to transfer across the placenta and the consequences to the offspring's health are still unclear [40]. One study conducted in the United States showed an increase of 5.6% in the use of antidepressants during pregnancy between 1998 and 2005, from 2.5% to 8.1%; with SSRIs being the most commonly used group of drugs (ranging between 71% [39] and 84% [8]). Taking into account the increased tendency in antidepressant use, our frequency of reported maternal use is consistent with the literature. Further, the 15% prevalence of depression in our study is slightly higher than the previously reported 9.1% to 14%; this variation depends on the study design and the trimester of pregnancy [41,42,7]. Our slightly higher prevalence may be due, in part, to the fact that depression frequencies vary by smoking during pregnancy [43,44]. Thirty-three percent of smoking mothers were depressed, while 10.7% of the non-smoking mothers reported depression. Besides the harmful effects of smoking, it is still unclear how the fetus reacts to maternal mood changes or intake of antidepressants during pregnancy. As a consequence, research in the field of maternal depression and associated drug use is gaining in importance.

We analyzed the possible association between maternal depression and changes in DNA methylation level at the imprinted control domains of *IGF2 *(*IGF2 *DMR and *H19 *DMR) and did not observe any differences between newborns exposed to maternal depression and individuals from mothers without any reported depression during pregnancy. However, earlier studies conducted in predominantly Caucasian populations suggested that the methylation status of key regulatory regions of certain genes may be sensitive to prenatal maternal mood, stress, or undernutrition [28,1]. A study in the senior offspring of mothers who were exposed to the Dutch famine of 1944 during periconception, as well as to the related emotional stress, showed a 5% lower mean methylation at the *IGF2 *DMR compared to the non-exposed same-sex sibling [1]; and Oberlander et al. reported that prenatal exposure to maternal depression or anxious mood is associated with increased methylation at a CpG-rich region of the *NR3C1 *gene [28]. Although exposure to SSRIs did not influence the methylation status of *NR3C1 *CpG sites in this latter study, animal models have shown that SSRIs may significant affect the expression level of this glucocorticoid receptor [45]. In our analysis on the methylation status at the *IGF2 *and *H19 *DMRs we did not detect an overall effect of antidepressant use, but when we looked at the interaction term between race and intake of antidepressants at any time during pregnancy (Table 3), and consecutively at the stratification by race (Table 4), our results suggest an association between African American mothers taking antidepressants and hypermethylation at the differentially methylated region of *IGF2 *upstream of *H19 *in the offspring. Hypermethylation at *H19 *DMR has been associated with a remarkable down-regulation of *H19 *expression, loss of imprinting of *IGF2*, and several disorders [46-48], including Wilm's tumors in children; a cancer type that is more prevalent in African Americans [49,50]. Race specific hypermethylation has been reported in a prostate cancer study where black men show a higher percentage of methylation on the CD44 promoter-region, compared to white men [51,52]; black men are almost twice as likely to exhibit CD44 hypermethylation compared to white men independent of tumor grade or disease stage [53]. A study on the potential epigenetic influences on racial disparity in the progression of endometrial cancer has also shown that cancers from black women demonstrate a significant lower ribosomal DNA methylation than tumors from white women [54]. The racial differences in methylation levels in these studies, as well as in our study, could reflect inherent genetic differences between Caucasians and African Americans, although we do not exclude influences of unmeasured race-related environmental factors. To our knowledge, a race-dependent effect of prenatal exposure to psychotropic medications on gene methylation has not previously been described. Race or ethnicity is still poorly documented in genetic cancer risk studies, especially in the field of epigenetics; and race is often not included in drug-related studies [55]. Considering the fact that pre-pregnancy BMI and education shared some effects on our DNA methylation outcomes, other factors not examined in this study, such as weight gain during pregnancy, specific dietary patterns, or other life-style factors, should be considered in the future. These, or other yet unidentified epigenetic determinants, may possibly explain the racial discrepancy we observe.

The present study has several limitations. The NEST population is hospital based and limited to pregnant women visiting Hospitals affiliated to Duke University. Although, home birth and home consults are very uncommon in the U.S.. Eighteen percent of the mothers refused to participate, and for practical reasons our analysis was focused on the first 436 participants only. We verified the distributions of the characteristics in all groups (with and without refusal/exclusions) and did not see any significant changes in distributions. Our study population is representative for pregnant mothers from Caucasian or African American origin in Durham County, NC. We verified the population from NEST with the US Census in Durham County [56], and the distribution of maternal age in our study is similar to the American Community Survey Estimates (ACSE) (2005-2009), reporting the numbers of women with birth in the last 12 months. Although the ACSE data suggest equal proportions of Caucasian and African American women giving birth (38.8% and 37.4%, respectively), we have slightly more African American mothers (49.3%) participating the study than Caucasians (44.3%). The Census reports 23.8% of "other races or origin", while only 5.0% of the NEST participants report this. A possible explanation can be the restriction to English speaking women in our study design. When comparing education, the Census data suggest that 56.3% of the mothers have lower education, compared to 65.1% in NEST. However, adjusting for education and race in our models did not alter the results.

In our study, twenty percent of the mothers smoke during pregnancy. This is six percent higher than what is expected in the US [57]. As mentioned in our earlier published study [34], NEST originally focused on smokers, and was later expanded to all pregnant mothers (see Materials and Methods). As a consequence our high percentage of pregnant smokers is not representative of the general population and smoking is oversampled. In order to verify if possible bias from this study design would affect our results we did a sensitivity analysis and rerun our data without the first part of the cohort (where smoking mothers were focused) and found the same associations. We also verified the distributions of all variables in smoking mothers in both phases of recruitments, and found no significant differences frequencies of depression, antidepressant intake, race distribution, education, age, birth weight or BMI.

Further, while low birth weight has been associated with depression [11] or intake of antidepressants [41], we do not detect an association between depression and birth weight. In mothers taking antidepressants we see a slightly higher percentage (+6%) of newborns with a birth weight less than 2.5 kg, although the difference is not significant (p = 0.11). Another limitation in our study is the reliance on self-report to identify individuals with depression, despite our verification with medical records. We do not exclude the possibility that depression may be slightly underreported: people with depression may not always report their mood changes or be diagnosed with depression. Moreover, no data on duration, history, dosage or rating scales were collected to evaluate any dose-response patterns. In addition, the use of antidepressants was based on medical records without verification of dose-compliance from the pregnant women. Further, only 45.5% of the mothers taking antidepressants report depression (Table 1). The reason for this could be attributed to the following: psychotropic drugs are widely prescribed for reasons other than depression [58], and as earlier mentioned the prevalence of depression may be underestimated. Nevertheless, our data suggest that methylation was not affected by the reported depression, while the effect of psychotropic drugs remained significant. As a consequence, the intake of antidepressants in African American pregnant women may be an independent predictor of hypermethylation at *H19 *DMR. We do not exclude a similar effect at the *IGF2 *DMR, given the small sample size of mothers taking antidepressants at this region studied. Further, we did not stratify by the class of antidepressants women used, given the low numbers, but the proportions of mothers taking SSRIs in both, African American and Caucasian, did not differ significantly and was relatively high: 71.4% of the African American mothers and 75.9% of the Caucasian mothers took SSRIs (p = 0.75). A larger study is warranted to understand more about the possible effects of antidepressant use in pregnancy. In conclusion, we report a race-dependent association between maternal use of antidepressants and hypermethylation at at least one of the imprint regulatory regions of *IGF2 *in the offspring. Although we do not know the exact underlying cause, infants born to African American mothers using antidepressants in pregnancy suggest an adverse effect on the methylation status of the *H19 *DMR, indicating a higher risk for loss of imprinting of *IGF2 *and potentially pernicious consequences for their health status in later life.

## Competing interests

The authors declare that they have no competing interests.

## Authors' contributions

AS designed the presented study, performed the statistical analysis, and wrote the manuscript. SKM co-conceived NEST, directed laboratory data acquisition and analyses, and contributed to the editing process of the manuscript. ZH generated data and oversaw laboratory analyses. AM oversaw participant recruitment in clinic. JMS contributed to the logistics of the data collection, interpretation of the results and editing of the manuscript. RLJ contributed to the NEST design and hypothesis. FW performed a second and independent data analysis to confirm the results. JK participated in the design of NEST. WDW contributed to the inception of the research hypothesis and aims, and contributed to draft the manuscript. MRF participated in the study design and helped editing the manuscript. CH conceived NEST, directed the data collection, the interpretations of the data, and contributed to draft the manuscript. All authors read and approved the final manuscript.
